# Functional specialization and differential regulation of short-chain carboxylic acid transporters in the pathogen *Candida albicans*

**DOI:** 10.1111/j.1365-2958.2009.07003.x

**Published:** 2009-12-24

**Authors:** Neide Vieira, Margarida Casal, Björn Johansson, Donna M MacCallum, Alistair JP Brown, Sandra Paiva

**Affiliations:** 1 Department of Biology, Molecular and Environmental Biology Centre (CBMA), University of Minho4710-057 Braga, Portugal; 2 Aberdeen Fungal Group, School of Medical Sciences, University of Aberdeen, Institute of Medical SciencesForesterhill, Aberdeen AB25 2ZD, UK

## Abstract

The major fungal pathogen *Candida albicans* has the metabolic flexibility to assimilate a wide range of nutrients in its human host. Previous studies have suggested that *C. albicans* can encounter glucose-poor microenvironments during infection and that the ability to use alternative non-fermentable carbon sources contributes to its virulence. *JEN1* encodes a monocarboxylate transporter in *C. albicans* and we show that its paralogue, *JEN2,* encodes a novel dicarboxylate plasma membrane transporter, subjected to glucose repression. A strain deleted in both genes lost the ability to transport lactic, malic and succinic acids by a mediated mechanism and it displayed a growth defect on these substrates. Although no significant morphogenetic or virulence defects were found in the double mutant strain, both *JEN1* and *JEN2* were strongly induced during infection. Jen1-GFP (green fluorescent protein) and Jen2-GFP were upregulated following the phagocytosis of *C. albicans* cells by neutrophils and macrophages, displaying similar behaviour to an Icl1-GFP fusion. In the murine model of systemic candidiasis approximately 20–25% of *C. albicans* cells infecting the kidney expressed Jen1-GFP and Jen2-GFP. Our data suggest that Jen1 and Jen2 are expressed in glucose-poor niches within the host, and that these short-chain carboxylic acid transporters may be important in the early stages of infection.

## Introduction

The opportunistic commensal organism, *Candida albicans,* is part of the normal gastrointestinal microflora in healthy individuals. *C. albicans* is a common cause of mucosal infections in immunodefective individuals, can be responsible for life-threatening systemic infections, in severely immunocompromised patients, and remains a common cause of nosocomial bloodstream infections in humans (Odds, 1988; Calderone, 2002; Perlroth *et al.*, 2007). As well as defective immune defences, a number of additional risk factors have been associated with invasive *C. albicans* infections. These include the use of broad spectrum antibiotics, the application of catheters or prosthetic devices, and the disruption of normal skin barriers (for a review see Perlroth *et al.*, 2007).

The pathogenicity of *C. albicans* is promoted by virulence factors such as adhesins, secretion of hydrolytic enzymes, morphogenesis and phenotypic switching (Odds, 1988; Brown and Gow, 1999; Calderone and Fonzi, 2001; Calderone, 2002). In addition, fitness attributes, such as its robust stress responses and metabolic flexibility, promote the pathogenicity of *C. albicans* (Wysong *et al.*, 1998; Alonso-Monge *et al.*, 1999; Hwang *et al.*, 2002). This fungus exhibits a notable capacity to sense and adjust to environmental changes, such as alterations in nutrient availability and extracellular pH, allowing it to survive within diverse niches such as the skin, mucous membranes, blood and internal organs of its human host (Odds, 1988; Calderone, 2002). Analyses of genome-wide expression profiles of *C. albicans* cells undergoing phagocytosis by mammalian macrophages showed a reprogramming of basal metabolism, which occurs in two successive steps (Prigneau *et al.*, 2003; Lorenz *et al.*, 2004). The first step involved the upregulation of genes encoding gluconeogenic, glyoxylate cycle and fatty acid β-oxidation enzymes, and the concomitant downregulation of glycolytic genes. The upregulation of genes encoding key glyoxylate cycle enzymes was also observed when *C. albicans* cells were exposed to human blood and neutrophils (Rubin-Bejerano *et al.*, 2003; Fradin *et al.*, 2005). The second step occurred as *C. albicans* cells escaped from macrophages and was coincident with the morphogenetic switch from the budding yeast form to the hyphal form. This late response was characterized by a resumption of glycolysis and a derepression of the translation machinery. The early response appears to be a major feature of the adaptation to non-fermentable carbon sources and was presumed to reflect glucose deprivation. *C. albicans* mutants that lack isocitrate lyase (*icl1*) display attenuated virulence in a mouse model of systemic candidiasis (Lorenz and Fink, 2001; Barelle *et al.*, 2006). This suggests a requirement for the synthesis of C4 compounds from acetyl-CoA, at some stage in the infection process (Lorenz and Fink, 2001; Lorenz and Fink, 2002). Because the early response also included the upregulation of genes involved in lipid degradation, it was postulated that lipids could be the precursors of acetyl-CoA, and thus that peroxisomal fatty acid β-oxidation is important for fungal virulence (Lorenz and Fink, 2002).

The use of green fluorescent protein (GFP) fusions to monitor gene activity has reinforced these findings. Using this approach it was shown that glyoxylate cycle and gluconeogenic genes are induced in individual *C. albicans* cells following phagocytosis by macrophages and neutrophils, but not during cell-to-cell contact (Barelle *et al.*, 2006). However, it has now been shown that fatty acids are not the only sources of acetyl-CoA available to *C. albicans in vivo*. A *pex5* mutant, which is impaired in peroxisomal protein import and hence in fatty acid β-oxidation, is still virulent in the mouse model of systemic candidiasis (Piekarska *et al.*, 2006). Therefore it was postulated that this fungus uses non-fermentable carbon sources, such as acetate or lactate, to survive within the glucose-poor environment of the phagolysosome (Piekarska *et al.*, 2006). This idea was also supported by Ramirez and Lorenz (2007) who reported that the ability to assimilate alternative non-fermentable carbon sources contributes to the virulence of *C. albicans*. They showed that *C. albicans* requires carnitine acetyltransferases of the carnitine shuttle for growth on acetate as sole carbon source (Prigneau *et al.*, 2003; Strijbis *et al.*, 2008; Zhou and Lorenz, 2008).

The transport of carboxylic acids across the plasma membrane presumably is essential for the assimilation of these alternative carbon sources. These substrates are weak organic acids that partially dissociate in aqueous solution, according to their pKa(s) and to the pH value of the medium. The uptake of the undissociated form of these compounds can occur through the plasma membrane by a simple diffusion. However at pH of above 5, the carboxylic acids are predominantly present in their anionic form and their assimilation depends on transporter-mediated uptake (for a review see Casal *et al.*, 2008). In *Saccharomyces cerevisiae* it has been reported that the Fps1 channel promotes the facilitated diffusion of the undissociated form of acetic acid at low pH (4.5) (Mollapour and Piper, 2007), and the ScJen1 transporter is responsible for the active transport of lactate and pyruvate (Casal *et al.*, 1999; Akita *et al.*, 2000). *C. albicans* Jen1 was identified by sequence homology with ScJen1 (Casal *et al.*, 1999) and it was subsequently characterized as a lactate, pyruvate, propionate/proton symporter in this pathogen (Soares-Silva *et al.*, 2004). *JEN1* is upregulated about fivefold when *C. albicans* cells are phagocytosed by macrophages (Piekarska *et al.*, 2006). Similarly, *CYB2,* which encodes an l-lactate dehydrogenase, is upregulated following macrophage attack (Piekarska *et al.*, 2006), reinforcing the idea that carboxylate assimilation is an integral part of the *C. albicans* response to phagocytosis.

A recent study based on synteny analysis, sequence similarity and motif analysis revealed the existence of at least 35 fungal homologues of the *S. cerevisiae JEN1* gene in 13 different *Hemiascomycetes* and four *Euascomycetes* (Lodi *et al.*, 2007). A phylogenetic tree of ScJen1p homologues (Lodi *et al.*, 2007) showed the existence of two main clusters. The first cluster represents a Jen1 group of proteins that have been functionally characterized as monocarboxylate transporters. The second cluster comprises Jen2-like proteins. This cluster includes a new Jen1-like protein from *C. albicans,* Jen2, which has not been functionally characterized. In addition, the cluster contains KlJen2, which is a succinate and D,L-malate plasma membrane transporter in *Kluyveromyces lactis* (Lodi *et al.*, 2004; Queiros *et al.*, 2007). *Schizosaccharomyces pombe* also expresses a dicarboxylate transporter, but the Mae1 protein has no significant homology to the Jen protein family. Mae1 transporter is constitutively expressed and not subjected to glucose repression (Grobler *et al.*, 1995).

*Candida albicans JEN2* is closely related to *JEN1* (Casal *et al.*, 2008). Furthermore, *JEN2* is strongly upregulated during phagocytosis by macrophages (160-fold, compared with fivefold for *JEN1*) (Piekarska *et al.*, 2006), and is strongly repressed by glucose (18-fold). Therefore, we reasoned that *JEN2* might play a significant role in the assimilation of short-chain carboxylic acids by *C. albicans.* We have confirmed this, showing that Jen2 executes a distinct function from Jen1 in *C. albicans*. Jen2 is a plasma membrane transporter, rapidly degraded following exposure to glucose, responsible for the saturable kinetics observed for malate and succinate uptake. Given the importance of non-fermentable carbon sources to the growth of *C. albicans* in some host niches (Prigneau *et al.*, 2003; Strijbis *et al.*, 2008; Zhou and Lorenz, 2008) and the potential importance of short-chain carboxylates as a carbon source *in vivo,* we have also compared the expression of Jen1 and Jen2 in disease models.

## Results

### *JEN2* encodes a dicarboxylate transporter in *C. albicans*

The *C. albicans JEN2* (*orf19.5307*) *locus* was annotated as a putative carboxylic acid plasma membrane transporter on the basis of its sequence similarity to *ScJEN1* (http://www.candidagenome.org/). However, Jen2 function has not been tested experimentally. There is only one *JEN locus* in *S. cerevisiae,* even though this species has undergone whole genome duplication (Scannell *et al.*, 2007). Yet there are two *JEN loci* in *C. albicans,* although this pathogen has not undergone whole genome duplication. We reasoned therefore, that the functions of *C. albicans JEN1* and *JEN2* might differ in *C. albicans.* Consequently, to determine the physiological role of *JEN2* in *C. albicans*, both chromosomal copies of this gene were disrupted in the RM1000 strain, resulting in strain CNV3 (*jen2*Δ/*jen2*Δ: Table 1). Additionally, a double *jen1jen2* mutant was constructed, resulting in *C. albicans* strain CNV4 (*jen1*Δ/*jen1*Δ, *jen2*Δ/*jen2*Δ: Table 1).

**Table 1:** *Saccharomyces cerevisiae* and *Candida albicans* strains used in this study.

Strain	Genotype	Reference
*S. cerevisiae*		
W303-1A	*MATa ade2 leu2 his3 trp1 ura3*	Thomas and Rothstein, 1989
BLC 491-U2	*MATa ura3-52 JEN1: :GFP Kanr*	Paiva *et al.*, 2002
*jen1*Δ	*W303-1A jen1*::KanMx4	Paiva *et al.*, 2004
*jen1*Δ– p*416GPD*	*jen1*Δ*transformed with* p*416GPD*	Soares-Silva *et al.*, 2004
*jen1*Δ*–*p*416GPDCaJEN2*	*jen1*Δ*transformed with* p*416GPDCaJEN2*	This work
*C. albicans*		
RM1000	*ura3::imm434/ura3::imm434, his1::hisG/his1::hisG*	Negredo *et al.*, 1997
CPK2	*ura3::imm434/ura3::imm434 his1::hisG/his1::G jen1::HIS1/jen1::URA3*	Soares-Silva *et al.*, 2004
CNV3	*ura3::imm434/ura3::imm434 his1::hisG/his1::G jen2::HIS1/jen2::URA3*	This work
CNV4	*ura3::imm434/ura3::imm434 his1::hisG/his1::G jen1::HIS1/jen1::ura3-, jen2::ura3-/jen2::URA3*	This work
CNV2-1	CPK2 with *RPS1-*CIp20	This work
CNV2-2	CPK2 with *RPS1-*CIp20-*JEN1*	This work
CNV3-1	CNV3 with *RPS1-*CIp20	This work
CNV4-1	CNV4 with *RPS1-*CIp20	This work
CNV4-1	CNV4 with *RPS1-*CIp20-*JEN1*	This work
CPK20-5	RM1000 with *JEN1/JEN1-GFP-URA3*	Soares-Silva *et al.*, 2004
CNV30-5	RM1000 with *JEN2/JEN2-GFP-URA3*	This work
CJB	*ura3::λimm434/ura3::λ imm434, pACT1-GFP*	Barelle *et al.*, 2004
CJB-1	*ura3::λimm434/ura3::λ imm434,* pGFP	Barelle *et al.*, 2004
CJB-3	*ura3::λimm434/ura3::λ imm434*, *pICL1GFP*	Barelle *et al.*, 2004

**Fig. 1:**
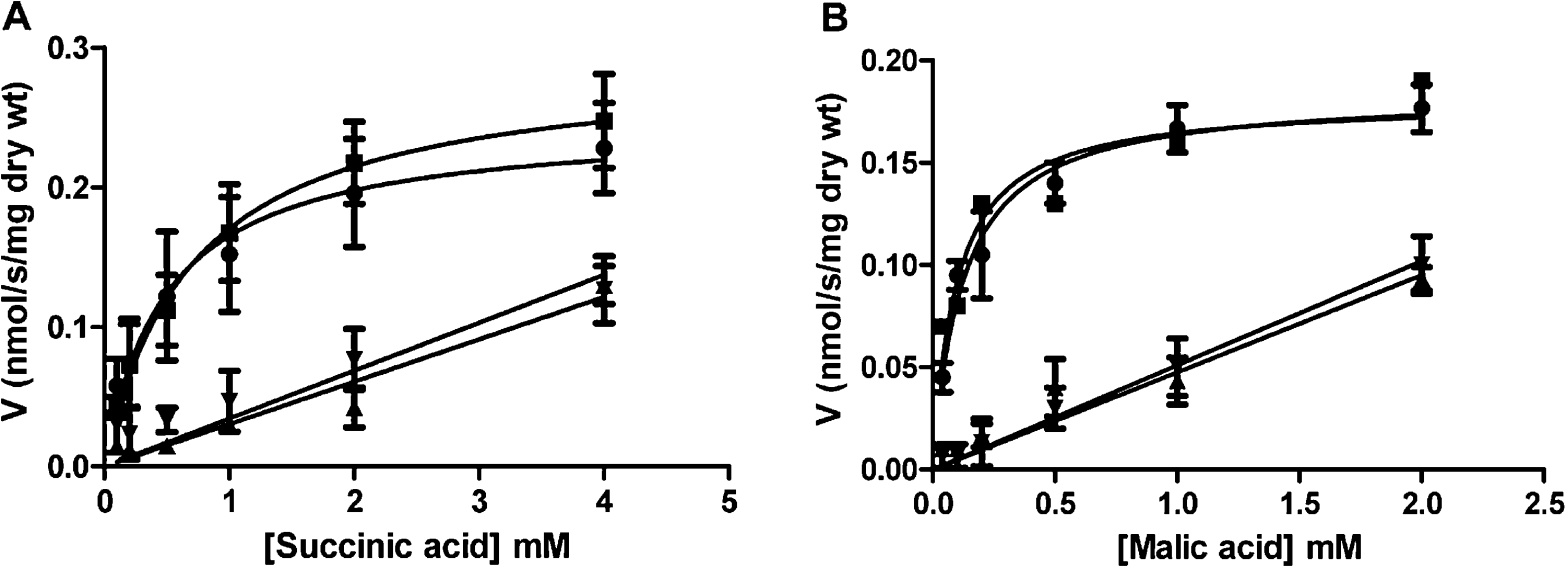
Transport of carboxylic acids in *Candida albicans*. A.Initial uptake rates of [2,3-^14^C] succinic acid, at pH 5.0, as a function of succinic acid concentration, after growth in medium containing succinic acid: RM1000 (wild-type), •; CPK2 (*jen1*), 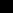; CNV3 (*jen2*), 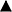; CNV4 (*jen1jen2*), 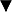. B.Initial uptake rates of L-[1,4(2,3)-^14^C] malic acid, at pH 5.0, as a function of malic acid concentration after growth in medium containing malic acid: symbols as for (A).

The ability of these new *jen2* and *jen1jen2* mutants to take up succinic acid and l-malic acid was compared with wild-type and *jen1* cells. We examined the uptake of succinic acid and malic acid because *JEN2* is found in the same phylogenetic cluster as *KlJEN2,* which is a succinate and malate transporter in *Kluyveromyces lactis* (Lodi *et al.*, 2004; Queiros *et al.*, 2007). Cells were grown in succinic acid and malic acid containing medium, at pH 5.0, under derepressing conditions (i.e. in the absence of glucose), and then the initial uptake rates of labelled succinic acid and l-malic acid were measured. Computer-assisted non-linear regression analyses to the experimental data suggested the presence of a mediated transport system for succinic acid in wild-type *C. albicans* cells (RM1000: Table 1) with the following kinetic parameters: *K*_m_ 0.49 ± 0.27 mM and *V*_max_ 0.25 ± 0.040 nmol s^−1^ mg dry wt.^−1^ (for concentrations between 0.1 mM and 4 mM, pH 5.0) (Fig. 1A). Malic acid uptake by wild-type cells also displayed saturable kinetics with a *K*_m_ of 0.12 ± 0.019 mM, and a *V*_max_ of 0.18 ± 0.0070 nmol s^−1^ mg dry wt.^−1^ (for concentrations between 0.04 mM and 2 mM, pH 5.0) (Fig. 1B).

Similar observations were made for *C. albicans jen1* mutant (CPK2: Table 1). For succinic acid transport, this mutant displayed a *K*_m_ of 0.71 ± 0.27 and a *V*_max_ of 0.29 ± 0.038 nmol s^−1^ mg dry wt.^−1^ (Fig. 1), and these *jen1* cells transported malic acid with a *K*_m_ of 0.096 ± 0.033 mM and a *V*_max_ of 0.18 ± 0.015 nmol s^−1^ mg dry wt.^−1^ (Fig. 1). In contrast, the uptake of succinic and malic acid by *jen2* and *jen1jen2* cells fitted to a first order kinetics (Fig. 1). These results show that *JEN2* codes for a saturable (second order kinetics) transport system of succinic and malic acid across the plasma membrane in *C. albicans*. In contrast, the kinetics of lactic acid transport was not affected by deletion of *JEN2*, but fitted to a first order kinetics in the strains lacking *JEN1* (not shown). Therefore, the Jen1 and Jen2 transporters have different specificities in *C. albicans*: Jen1 transports short-chain monocarboxylic acids such as lactate, whereas Jen1 transports short-chain dicarboxylic acids such as succinate and malate.

### *jen1jen2* mutant is affected in the growth on mono- and dicarboxylic acids

**Fig. 2:**
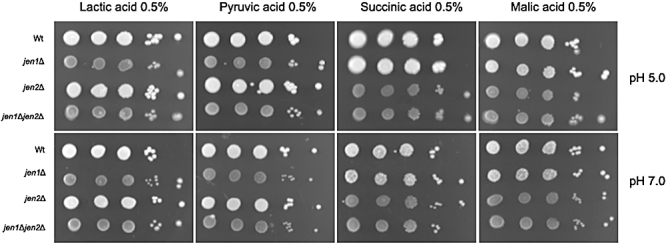
Growth phenotypes, at 37°C, of *Candida albicans* RM1000 (wild-type), CPK2 (*jen1*), CNV3 (*jen2*), CNV4 (*jen1jen2*), incubated for 96 h, in the following solid media: SC-lactic acid (0.5%, w/v, pH 5.0 or 7.0); SC-pyruvic acid (0.5%, w/v, pH 5.0 or 7.0); SC-succinic acid (0.5%, w/v, pH 5.0 or 7.0); SC-malic acid (0.5%, w/v, pH 5.0 or 7.0). Cells were serially diluted; 3 µl of drops of each dilution were spotted onto the plates.

Given that *C. albicans jen* mutants have defects in carboxylic acid transport, it was conceivable that these mutants might also display growth defects on the corresponding carbon sources. Therefore, the growth of wild-type and *jen* mutants was evaluated on solid media containing different carbon and energy sources, such as glucose, lactic, pyruvic, malic and succinic acids. Growth was evaluated at pH 5.0 and pH 7.0, and the cells were incubated across a range of temperatures: 18°C, 30°C and 37°C. When incubated at 37°C, for 96 h, *jen1* and *jen1jen2* mutants displayed a growth defect on media containing lactic or pyruvic acids, as sole carbon source (Fig. 2). The *jen2* and *jen1jen2* mutants exhibited a growth defect on succinic and malic acids (Fig. 2). The residual growth observed for the mutant strains could be attributed to simple diffusion; however, because it also persisted in the media with pH 7.0, it is also likely due to the presence of other transport systems, not identified yet.

### Heterologous expression of *C. albicans JEN2* in *S. cerevisiae*

**Fig. 3:**
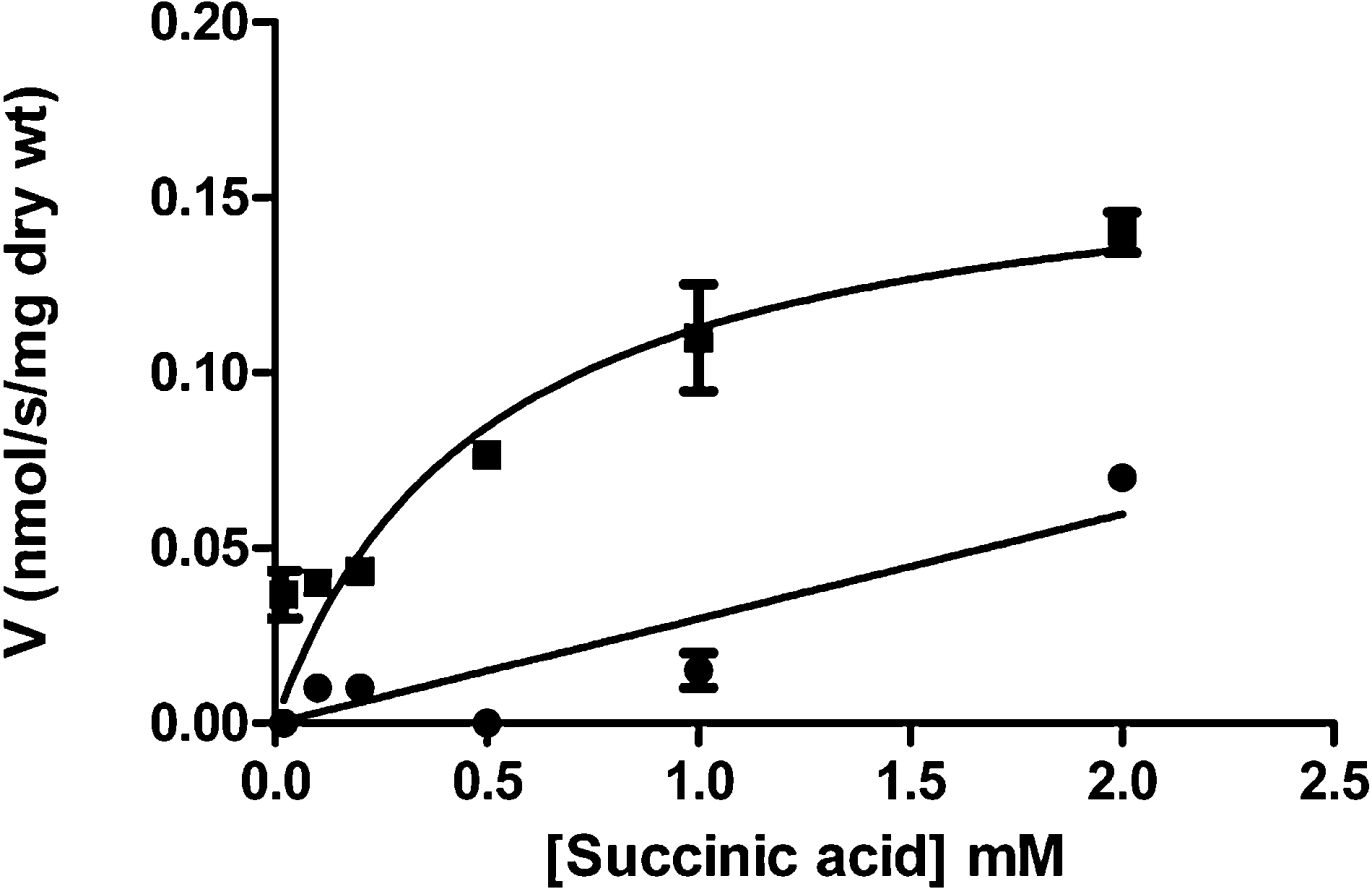
Heterologous expression of *Candida albicans JEN2* in *Saccharomyces cerevisiae.* Initial uptake rates of [2,3-^14^C] succinic acid at pH 5.0, as a function of succinic acid concentration, by YNB-glucose-grown cells of *S. cerevisiae* W303-1A *jen1Δ* strain transformed with p416GPD (•) or pNV3 (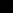).

No transporters for dicarboxylic acids have been so far assigned in *S. cerevisiae,* being assumed that these substrates cross the plasma membrane by a simple diffusion mechanism of the undissociated molecules (Salmon, 1987; Queirós *et al*., 2003). *S. cerevisiae jen1* cells that express *KlJEN2* in the plasmid p416-GPD (glyceraldehyde 3-phosphate dehydrogenase) acquire the ability to transport succinic and malic acids by a mediated mechanism (Queiros *et al.*, 2007). Therefore *C. albicans JEN2* was cloned into the same *S. cerevisiae* expression vector, p416-GPD (Mumberg *et al.*, 1995), resulting in the plasmid pNV3 (Table 2). *S. cerevisiae jen1* cells were transformed with this plasmid (pNV3) and with the control empty vector (p416-GPD: Table 2). We then determined the kinetic parameters for succinate uptake at pH 5.0 by these strains. *S. cerevisiae jen1* cells expressing *C. albicans JEN2* acquired the ability to transport succinate by a mediated mechanism with a *V*_max_ of 0.17 ± 0.020 nmol s^−1^ mg dry wt.^−1^ and a *K*_m_ of 0.50 ± 0.16 mM (Fig. 3). In contrast, control cells containing p416-GPD behaved as the wild-type. The kinetic parameters obtained were equivalent to the ones found in *C. albicans* wild-type cells. These data confirm the function of *C. albicans* Jen2 as a dicarboxylate transporter.

**Table 2:** Plasmids used in this study.

Plasmids	Source or reference
CIp20	Murad *et al.*, 2000
pNV1 (*CaJEN1* in Cip20)	This work
pGFP-URA3	Gerami-Nejad *et al.*, 2001
pLUL	Dennison *et al.*, 2005
pLHL	Dennison *et al.*, 2005
pDDB57	Wilson *et al.*, 2000
p416GPD	Mumberg *et al.*, 1995
pNV3 (*CaJEN2* in p416GPD)	This work

### Expression of *JEN1/JEN2* and subcellular localization of Jen1-GPF and Jen2-GFP in living *C. albicans* cells in response to different carbon sources

**Fig. 4:**
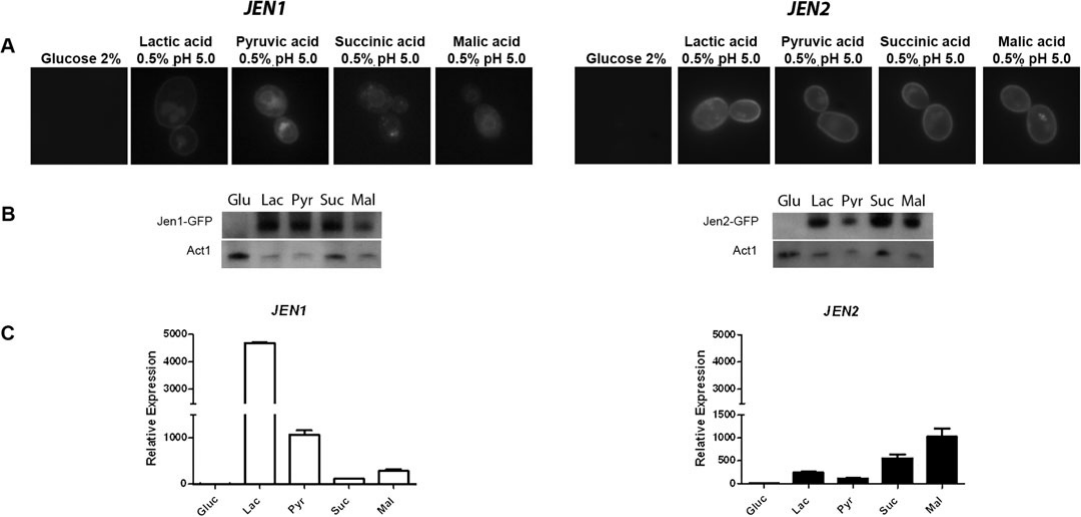
Expression of *JEN1/JEN2* and localization of Jen1-GFP and Jen2-GFP in living *Candida albicans* cells grown on different carbon sources. Mid-exponential *C. albicans* cells grown in minimal medium containing 2% w/v glucose were washed twice with deionized water and then transferred, for 4 h at 30°C, to fresh minimal media containing different carbon sources: glucose 2%, w/v (glu); lactic acid 0.5%, v/v (lac); pyruvic acid 0.5%, v/v (pyr); succinic acid 0.5%, v/v (suc); malic acid 0.5%, v/v (mal). Samples were collected after induction, to examine cells by epifluorescence microscopy, to pepare mRNA and protein extracts. A.Subcellular localization of Jen1-GFP and Jen2-GFP in living cells. B.Protein extracts were separated by SDS-PAGE and analysed for Jen1-GFP and Jen2-GFP by western immunoblotting with an anti-GFP antibody. The blots were reprobed with an anti-ACT1 to provide loading controls. C.Expression analysis of *JEN1* and *JEN2* was followed by qRT-PCR. *JEN1* and *JEN2* mRNA expression levels were normalized to *ACT1*. The results are presented as the mean ± SD of two independent experiments with duplicates for each experiment.

*Candida albicans* Jen2 was tagged with the reporter gene *GFP* at its carboxy-terminus (Table 1) to study the expression and sub-cellular localization of this transporter. The behaviour of this Jen2-GFP fusion in *C. albicans* was compared with a previously constructed Jen1-GFP fusion (Table 1). Cells expressing Jen1-GFP and Jen2-GFP were grown in minimal medium supplemented with 2% glucose to mid-exponential phase. Cells were then washed and transferred to fresh minimal media containing different carbon sources. After 4 h of incubation, at 30°C, samples were collected to examine cells by epifluorescence microscopy, to prepare extracts for further analyses by western immunoblotting with anti-GFP antibody and to prepare mRNA for quantitative real-time polymerase chain reaction (qRT-PCR) studies. The results show that the Jen2-GFP fusion was expressed and mainly localized to the plasma membrane, after derepression in lactic, succinic, pyruvic and malic acids (Fig. 4A), although some intracellular fluorescence was also observed. The levels of Jen2 protein expression were measured by Western blot analyses with an anti-GFP antibody, and compared with the internal control, Act1. The Jen2-GFP signal was detected in all of the conditions tested, except in glucose grown cells (Fig. 4B), which was consistent with the fluorescence data. We also quantified *JEN2* mRNA levels by qRT-PCR (Fig. 4C). The data show lower *JEN2* mRNA levels during growth on lactic or pyruvic acid compared with succinic or malic acid grown cells. This result correlates reasonably well with the levels of the corresponding Jen2-GFP fusions (Fig. 4B and C), although some post-translational regulation of Jen2-GFP levels cannot be excluded.

With respect to Jen1-GFP subcellular expression, the images show a faint plasma membrane localization in lactic and pyruvic acid derepressed cells, although some intracellular fluorescence was also detected (Fig. 4A). Very low Jen1-GFP fluorescence levels were observed in malic and succinic acid derepressed cells. This was consistent with the Jen1-GFP signals on Western blots, which were lower for malic and succinic acid-derepressed cells relative to the Act1 loading control (Fig. 4B). Furthermore *JEN1* transcript levels were lower in these cells compared with those in cells derepressed on lactic or pyruvic acids (Fig. 4C). However, in lactic and succinic acids derepressed cells there appeared to be a lack of correlation between the levels of *JEN1* mRNA and Jen1-GFP protein (Fig. 4B and C) in that the relatively high levels of *JEN1* mRNA in lactic acid were not reflected in equivalent Jen1-GFP levels; also the signal obtained for Jen1-GFP by Western blot in succinic acid was not consistent with the very low levels of *JEN1* mRNA and the weak fluorescence found (Fig. 4A–C). Similar observations were reproduced in three independent experiments. We have, previously, confirmed that Jen1 fused with GFP is a functional lactate transporter with identical *K*_m_ and *V*_max_ as the wild-type. Therefore, the GFP fusion does not seem to affect the folding and/or localization of Jen1. This apparent inconsistency between *JEN1* mRNA and proteins levels in lactic and succinic acid-grown cells could be explained by post-translational control of Jen1 expression levels, but this remains to be tested. Nevertheless, we have already shown that post-translational control mechanisms exist at the level of Jen1/Jen2 protein turnover in response to glucose (Andrade and Casal, 2001; Paiva *et al.*, 2002; Queirós *et al.*, 2003).

Taken together these results indicate that monocarboxylic acids induce *JEN1* expression to a greater degree than *JEN2*, whereas *JEN2* is induced more strongly than *JEN1* during growth on dicarboxylic acids. These data, which suggest that the Jen transporters play distinct physiological roles in *C. albicans* cell, are consistent with the results obtained from the transport and growth assays. It still remains to be explained why *JEN* genes and proteins are also expressed under conditions where they do not appear to be functional: Jen1 is expressed in the presence of dicarboxylic acids and Jen2 in monocarboxylic acids.

### Inactivation of Jen1-GFP and Jen2-GFP by glucose in *C. albicans*

**Fig. 5:**
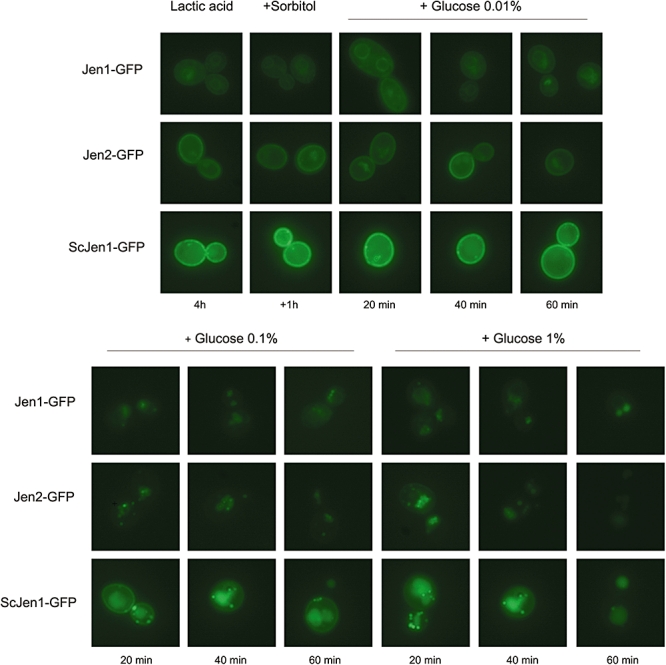
Timecourse of Jen1-GFP, Jen2-GFP and ScJen1-GFP inactivation at different glucose concentrations. Induced cells were treated with 0.01% (0.55 mM), 0.1% (5.5 mM) and 1% (55 mM) glucose or with sorbitol at the same concentrations (as a control for osmotic shock) and examined by fluorescence microscopy, after continued incubation: CPK20-5 (*Candida albicans JEN1-GFP*); CNV30-5 (*C. albicans JEN2-GFP*); BLC 491-U2 (*Saccharomyces cerevisiae JEN1-GFP*).

Jen proteins are generally subjected to tight glucose repression (Paiva *et al.*, 2002; Lodi *et al.*, 2004; Queiros *et al.*, 2007; Soares-Silva *et al.*, 2007). In *S. cerevisiae* the addition of glucose to lactic acid-grown cells triggers the loss of ScJen1p activity and the repression of *ScJEN1* expression (Andrade and Casal, 2001; Paiva *et al.*, 2002). The loss of ScJen1-GFP is the result of its End3-dependent internalization and its subsequent targeting to the vacuole for degradation (Paiva *et al.*, 2002). This glucose-regulated endocytosis of ScJen1 in *S. cerevisiae* has been characterized in detail and it is one of the few examples for which ubiquitin-K63 linked chain(s) have been shown to be required for correct trafficking at two stages of endocytosis: endocytic internalization and sorting at Multi Vesicular Bodies (MVBs) (Paiva *et al.*, 2009). To compare the impact of glucose upon *C. albicans* Jen proteins with ScJen1, *C. albicans* cells carrying the *JEN1-GFP* or *JEN2-GFP* fusions were compared with *S. cerevisiae* cells expressing *ScJEN1-GFP.* These strains were grown in minimal medium containing 2% glucose and then derepressed for 4 h in minimal medium containing 0.5% lactic acid at pH 5.0, a condition where all transporters were shown to be expressed and localized to the plasma membrane (Fig. 4A). These cells were then treated with glucose, at final concentrations ranging from 0.01% to 1%. These conditions were selected to cover the range of glucose concentrations found in human blood, which generally varies between 3 and 5 mM (equivalent to about 0.06–0.1% glucose) (Barelle *et al.*, 2006). Control cells were treated with sorbitol (final concentration 110 mM) as a control for the osmotic changes imposed by the glucose addition. After these treatments the cells were examined by epifluorescence microscopy (Fig. 5).

The first observation of note in these experiments was that fluorescence levels were generally higher in *S. cerevisiae* than *C. albicans* (Fig. 5). This was consistent with published comparisons of GFP levels in these two yeasts (Cormack *et al.*, 1997). The next observation was that even 1 h after the addition of 0.01% glucose to the *C. albicans* and *S. cerevisiae* cells, Jen1-GFP, Jen2-GFP and ScJen1-GFP levels persisted in their plasma membranes (Fig. 5). However, within 20 min of incubation with 0.1 or 1% glucose, all the Jen-GFP fluorescence in *C. albicans* cells had disappeared from the plasma membrane (Fig. 5). In the *S. cerevisiae* cells incubated with 0.1% glucose, not all of the ScJen1-GFP signal was removed from the plasma membrane, but punctuate structures were observed in the cytoplasm (Fig. 5). When *S. cerevisiae* cells were treated with 1% glucose the ScJen1-GFP fluorescence signal finally disappeared from the plasma membrane after 60 min of incubation (Fig. 5). This indicates that the Jen1 and Jen2 transporters are internalized more quickly in response to glucose in *C. albicans* than ScJen1 in *S. cerevisiae.* We set to determine the threshold glucose concentration which triggers *C. albicans* Jen degradation. Because the results showed that Jen1 and Jen2 respond similarly to glucose in *C. albicans*, we carried out a Western blot analysis to follow Jen2-GFP expression, over time, after a pulse of 0.01% and 0.05% glucose, to lactic acid induced cells. In the presence of 0.05% (close to the minimal blood glucose concentration) the level of Jen2-GFP remained stable, even after 60 min of the pulse of glucose (data not shown). However, the addition of 0.1% glucose triggered Jen2-GFP degradation after 20 min of incubation. This indicates that Jen proteins may still be expressed in niches where only low amounts of glucose are present (≤ 0.05%). This is consistent with the view that *C. albicans* cells may be able to utilize some alternative carbon sources in such microenvironments. This view is strengthened by the observation that gluconeogenic and glyoxylate cycle genes are expressed by some *C. albicans* cells infecting the kidney (Barelle *et al.*, 2006).

### The inactivation of *JEN1* or *JEN2* does not affect *C. albicans* morphogenesis

The ability of *C. albicans* to switch between hypha and yeast growth forms is considered to be a virulence attribute of this fungus (Berman and Sudbery, 2002). The hyphal form facilitates the adhesion and invasion of human tissues as well as the evasion of phagocytic cells. In contrast, the yeast form is better adapted to dissemination (Gow *et al.*, 2002). Hence the ability to switch between morphological forms is important for *C. albicans* virulence (Berman and Sudbery, 2002; Gow *et al.*, 2002). Environmental pH is a trigger for *C. albicans* morphological differentiation (Buffo *et al.*, 1984) and influences nutrient uptake via the functionality of plasma membrane transporters. The responses of *C. albicans* to changes in extracellular pH have been analysed by global expression analysis, revealing links between extracellular pH and iron acquisition (Bensen *et al.*, 2004), another virulence determinant. Furthermore, the activities of plasma membrane proton transport systems are important in controlling internal pH and these are also associated with the regulation of dimorphism (Stewart *et al.*, 1988; 1989; Kaur and Mishra, 1994). The yeast form is favoured at low ambient pHs, for example around pH 4.0 when undissociated forms of carboxylic acids predominate and the simple diffusion of these acids into the cell is favoured. On the other hand, hyphal growth is stimulated at neutral ambient pH, when the mediated transport systems are more active (Buffo *et al.*, 1984; El Barkani *et al.*, 2000). Therefore, environmental pH, morphogenesis and carboxylic acid uptake are linked, a reason why we tested whether the inactivation of *JEN1* or *JEN2* affects morphogenesis in *C. albicans.*

Morphogenesis can be triggered by several different treatments such as exposure of *C. albicans* cells to temperatures higher than 37°C, ambient pH above 6.5, serum and low concentrations of dissolved O_2_. Serum has been shown to be the strongest inducer of hyphal development, when combined with temperatures of 37°C. For that reason, *C. albicans* RM1000 (wild-type), CPK2 (*jen1*), CNV3 (*jen2*) and CNV4 (*jen1jen2*) cells were grown under conditions that normally induce *JEN1* and *JEN2* expression (minimal medium containing 0.5% lactic acid at pH 5.0). These cells were then transferred to hypha inducing conditions by supplementing the medium with 10% (v/v) fetal calf serum and incubating at 37°C. The morphology of cells in these cultures was then monitored after 1, 2 and 3 h. Filamentation was also assessed in solid media supplemented with 10% (v/v) fetal bovine serum (FBS). Both wild-type and mutant strains formed hyphae at similar rates (data not shown). To strengthen these findings *C. albicans* cells were also subjected to pH (Buffo *et al.*, 1984) and GlcNAc switch (Mattia *et al.*, 1982). Again, both at higher pHs and in the presence of poor carbon and nitrogen sources, at temperatures above 37°C, yeast to hypha transition was induced in a similar way in all the strains tested. Therefore, the Jen1 and Jen2 carboxylate transporters were not required for hyphal development under the conditions tested.

### Regulation of *JEN1* and *JEN2* following contact with the host

Human blood is a complex and hostile environment for microorganisms. *C. albicans* cells adapt rapidly to this hostile environment, displaying dramatic changes in its transcript profile (Fradin *et al.*, 2003). The first line of defence against *C. albicans* is provided by phagocytic cells such as macrophages and neutrophils (Lorenz and Fink, 2002). Therefore, we examined the impact of phagocytosis upon the expression of the *C. albicans* carboxylate transporters.

**Fig. 6:**
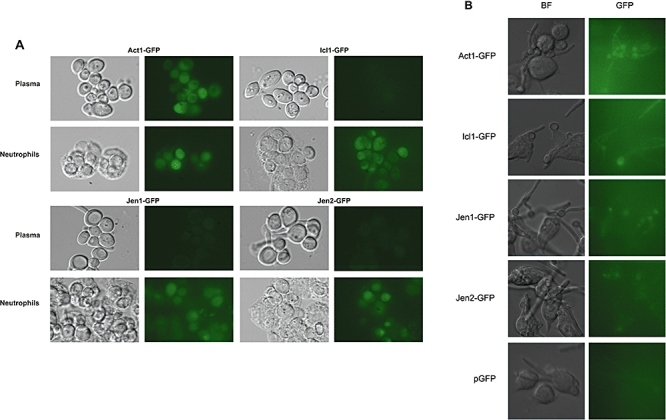
Differential regulation of Jen1-GFP and Jen2-GFP in *Candida albicans* following phagocytosis by immune cells. A.Interaction of *C. albicans* with neutrophils. *C. albicans* cells containing *ACT1-GFP, ICL1-GFP, JEN1-GFP, JEN2-GFP* or the control pGFP plasmid were mixed in a 1:1 ratio with human neutrophils, and examined microscopically after 2.5 h at 37°C. Corresponding light and fluorescence images are shown. B.Interaction of *C. albicans* with macrophages. Cultured murine macrophages (RAW.264.7) were mixed in a 1:3 ratio with the *C. albicans* strains, incubated for 3 h and analysed by light and fluorescence microscopy.

First we examined the behaviour of control *C. albicans* strains carrying pGFP, *ACT1-GFP* or *ICL1-GFP* fusions. The strains were incubated with murine macrophages or human neutrophils for 2.5–3 h at 37°C, and GFP expression was detected by fluorescence microscopy of the phagocytosed and non-phagocytosed *C. albicans* cells. All of the control strains behaved as expected (Fig. 6). No GFP fluorescence was detected in phagocytosed or non-phagocytosed cells in the negative control (pGFP). In contrast, the positive control (*ACT1-GFP*) was expressed under both conditions. Furthermore, *ICL1-GFP* was induced following phagocytosis, as reported previously (Lorenz *et al.*, 2004; Fradin *et al.*, 2005).

The fluorescence of *C. albicans* CPK20-5 (Jen1-GFP) and CNV30-5 (Jen2-GFP) cells was examined under equivalent conditions. Both Jen1-GFP and Jen2-GFP were expressed, although weakly, following phagocytosis by macrophages or neutrophils. In contrast, the non-phagocytosed cells in human plasma (a glucose-containing medium) displayed no significant Jen1-GFP and Jen2-GFP expression. As a further control, CPK20-5 and CNV30-5 cells were examined after 3 h of incubation at 37°C, 5% CO_2_, in glucose-containing cell culture medium [Dulbecco's modified Eagle's medium (DMEM)]. No fluorescence was observed (not shown). Therefore Jen1-GFP and Jen2-GFP expression mirrored that of the Icl1-GFP fusion. Expression of Jen1 and Jen2 carboxylate transporters was only observed following phagocytosis, presumably as a result of the transfer of *C. albicans* cells into the glucose poor environment of the phagolysosome (Fig. 6A and B).

If Jen1 and Jen2 are expressed following phagocytosis, do these carboxylate transporters promote the survival of phagocytosed *C. albicans* cells? We tested this by comparing the survival of *jen1* and *jen2* mutants with wild-type cells following exposure to human blood. RM1000 (wild-type), CPK2 (*jen1*), CNV3 (*jen2*) and CNV4 (*jen1jen2*) cells were grown to mid-exponential phase in minimal media at pH 5.0 containing either 0.5% lactic acid or 1% succinic acid. These mid-exponential cells were incubated with human blood for 30 min at 37°C, and then viable cell counts determined. As a negative control cells were plated after incubation in sterile water. In independent replicate experiments using blood from several different donors, we observed no significant difference in the blood killing between the wild-type cells and the *jen* mutant cells (60–65% survival). We conclude that the survival of phagocytosed *C. albicans* cells is not dependent upon Jen1 or Jen2.

### Single cell profiling of *C. albicans* carboxylate transporter genes during systemic infections and virulence of *C. albicans* null jen mutants

**Fig. 7:**
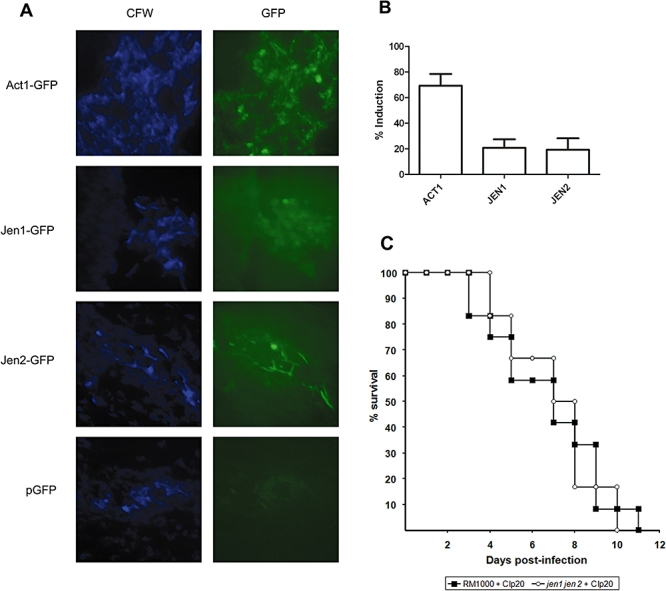
Expression of *Jen1-GFP* and *Jen2-GFP* in *Candida albicans* cells infecting the kidney. A.*C. albicans* cells infecting the kidneys of mice were visualized by staining with Calcofluor white (CFW), and then those that expressed their GFP fusions were imaged by fluorescence microscopy (GFP): Act1-GFP, Jen1-GFP and Jen2-GFP. B.The proportion of *C. albicans* cells infecting the kidney that display GFP fluorescence above background levels. C.Comparison of the virulence of wild-type and *jen1jen2* cells in the mouse model of systemic candidiasis.

After assessing the induction of the Jen1 and Jen2 carboxylate transporters in *ex vivo* infection models, we examined their expression *in vivo* in the mouse model of systemic candidiasis. Mice were infected with *C. albicans* strains containing *JEN1-GFP, JEN2-GFP, ACT1-GFP* or pGFP and humanely terminated after the animals lost approximately 20% of their body weight and/or showed signs of illness (3 or 4 days). The kidneys were removed aseptically and kidney sections prepared. Fungal cells in these sections were identified by Calcofluor white staining, and then GFP fluorescence in these cells detected by fluorescence microscopy (Gow and Gooday, 1982). As expected no GFP flucorescence was observed for the negative control (pGFP) (Fig. 7A). In contrast, over 80% of *ACT1-GFP* containing cells displayed fluorescence in the kidney sections. Jen1-GFP and Jen2-GFP were expressed in approximately 20–25% of the cells in infected kidneys (Fig. 7A and B). This is consistent with a previous report which suggested that about one-third of *C. albicans* cells infecting the mouse kidney assimilate carbon via gluconeogenesis (Barelle *et al.*, 2006). Interestingly, the intensity of Jen1-GFP and Jen2-GFP fluorescence was comparable *in vivo* and *in vitro* grown fungal cells, suggesting that in those *C. albicans* cells that activate *JEN1* and *JEN2* in the kidney, the level of *JEN* expression is similar to that observed in gluconeogenic cells *in vitro*.

Additionally, we determined the impact of *jen* mutations upon *C. albicans* virulence. Prototrophic wild-type cells and the *jen1jen2* double mutant were tested in a murine model of systemic candidiasis (MacCallum and Odds, 2005). No statistically significant differences in the virulence of the wild-type and *jen1jen2* cells were found. Furthermore similar fungal burdens were observed in the kidneys of infected animals. Therefore, the inactivation of Jen1 and Jen2 does not impair the virulence of *C. albicans*, at least in the mouse model of systemic candidiasis (Fig. 7C).

## Discussion

*Candida albicans* has the ability to survive within different host niches in part due to its metabolic flexibility. This versatile microorganism is a Krebs-positive yeast (Rao *et al.*, 1962) that can use different nutrients, including short-chain carboxylic acids, as sole carbon and energy sources. In this study we have shown that Jen2 is a dicarboxylate transporter that mediates the uptake of malate and succinate through the plasma membrane. This is in contrast to its *C. albicans* paralogue, Jen1, which transports lactate, pyruvate and propionatec (Soares-Silva *et al.*, 2004). This conclusion was based on three complementary observations. First, Jen2 localized to the plasma membrane (Fig. 4A). Second, the inactivation of *JEN2* (but not *JEN1*) was shown to abolish the saturable uptake of malic acid and succinic acid by *C. albicans* cells (Fig. 1) and to affect the growth in media containing those substrates, as sole carbon and energy sources (Fig. 2). Third, the heterologous expression of *C. albicans JEN2* in *S. cerevisiae* cells conferred upon them the ability to take up succinic acid by a Michaelis-Menten kinetics (Fig. 3). These experiments confirmed that *JEN1* and *JEN2* execute distinct transport functions in *C. albicans.*

The Jen1 and Jen2 transporters displayed distinct regulatory profiles in response to carbon source, both at the protein and at the mRNA level. Both were expressed during growth on lactic, pyruvic acid, succinic and malic acids (Fig. 4B and C). However, the monocarboxylic acids tested induced the expression of *JEN1* stronger than of *JEN2* whereas *JEN2* is more induced than *JEN1* in the presence of dicarboxylic acids (Fig. 4C). This differential regulation reinforces the notion that these transporters play distinct roles in *C. albicans.*

Both Jen1 and Jen2 are sensitive to glucose (Fig. 5). Following the addition of glucose at concentrations ≥ 0.1% to *C. albicans* cells growing on lactic acid, both Jen1-GFP and Jen2-GFP were rapidly internalized. Interestingly, the rates of Jen1-GFP and Jen2-GFP internalization in *C. albicans* were significantly faster than the rate of ScJen1-GFP internalization in *S. cerevisiae* (Fig. 5). Therefore, mechanistic differences might exist between *C. albicans* and *S. cerevisiae* with regard to the glucose mediated downregulation of Jen transporters in these yeasts. These results corroborate suggestions that *C. albicans* and *S. cerevisiae* may sense sugars differently (Brown *et al.*, 2006) despite both of these yeasts being sensitive to low concentrations of glucose (Rodaki *et al.*, 2009).

Considerable advances have been made in our understanding of how *C. albicans* responds to the metabolic stimuli encountered in its human host, but large gaps in our knowledge remain. Genome-wide expression profiling of *C. albicans* cells in *ex vivo* infection models have highlighted the significance of central metabolic pathways for the adaptation of this organism to its host (Lorenz and Fink, 2001; Lorenz and Fink, 2002; Fradin *et al.*, 2003), and it is now clear that these pathways are regulated in a niche-specific manner during infection (Barelle *et al.*, 2006). It has been postulated that glucose levels are low in the phagolysosome, but this microenvironment is rich in fatty acids and their products (Lorenz and Fink, 2001). These compounds may be sources of acetyl-CoA that feed the glyoxylate cycle (Lorenz and Fink, 2001; Lorenz *et al.*, 2004). However, acetate and lactate may be the main sources of acetyl-coA. This idea is consistent with microarray data, which reveal an upregulation of *JEN1* and *JEN2* in *C. albicans* cells phagocytosed by macrophages and neutrophils (Lorenz *et al.*, 2004; Fradin *et al.*, 2005; Piekarska *et al.*, 2006). Moreover, *S. cerevisiae* and *C. albicans* acetyl-CoA synthases, which are responsible for the conversion of acetate to acetyl-CoA, are upregulated (8.7- and 6.1-fold, respectively) after phagocytosis by macrophages and by neutrophils (Fradin *et al.*, 2005). It was suggested that acetate might be a product of lactate degradation that sustains *C. albicans* inside the phagolysosome (Piekarska *et al.*, 2006). Our results revealed that both Jen1-GFP and Jen2-GFP are expressed inside macrophages and neutrophils, but not in the bloodstream, a glucose-rich environment (Fig. 6). Moreover, in the murine model of systemic candidiasis about 20–25% of *C. albicans* cells infecting the kidney express Jen1 and Jen2 (Fig. 7), a similar value to Icl1 (Barelle *et al.*, 2006). These results support the idea that carboxylic acids such as lactate are present inside the phagolysosome, and suggest that both monocarboxylates and dicarboxylates, such as succinate and malate, may act as carbon sources that help to sustain *C. albicans* following phagocytosis. However, the inactivation of Jen1 and Jen2 does not attenuate the virulence of *C. albicans* (Fig. 7C). This result implies that maybe other, yet uncharacterized, carboxylate transporters play a role in the uptake of further carboxylic acids, such as acetic or citric acids.

The biochemical pathways that mediate monocarboxylate and dicarboxylate anabolism in *C. albicans* during the infection process remain to be confirmed. For example, it is not clear how malic acid is metabolized in this organism. According to the Candida Genome Database, *orf19.1867*, which displays homology to *S. pombe MAE1* (Grobler *et al.*, 1995), encodes a putative malate transporter that is induced during phagocytosis by macrophages (Prigneau *et al.*, 2003). Also, *orf19.3419* encodes a putative mitochondrial malic enzyme, with homology to *S. cerevisiae MAE1* (Boles *et al.*, 1998). This has resulted in a nomenclature conflict, because both *orf19.1867* and *orf19.3419* are referred to *MAE1* in *C. albicans*. In *S. pombe* the malic enzyme is encoded by *MAE2*, and it is a cytoplasmatic protein that, unlike ScMae1, has a high affinity for its substrate (Viljoen *et al.*, 1994). Our results suggest that those *C. albicans* genes currently presumed to be malate transporters are not involved in the uptake of dicarboxylic acids across the plasma membrane, because deletion of *JEN2* abolished the mediated uptake of succinate and malate under the conditions tested (Fig. 1). Nevertheless, it is conceivable that another malate transporter exists in *C. albicans*, with a different pattern of regulation. Alternatively, *C. albicans* might express other malic acid transporters at alternative cellular locations, for example in the peroxisome (Tournu *et al.*, 2005; Piekarska *et al.*, 2008).

The same intriguing questions arise for a malic acid enzyme, whose activity has been reported in *C. albicans in vitro* (Rao *et al.*, 1962). This enzyme converts malic acid into pyruvate. Depending upon yeast species and growth conditions, malic acid can be further converted into oxaloacetate and enter the Krebs cycle or can be metabolized to ethanol via acetaldehyde during maloalcoholic fermentation (Rodriguez and Thornton, 1990). *S. cerevisiae* can only use small amounts of malic acid under anaerobic conditions in the presence of glucose, by fermenting it to ethanol or to succinate via fumarate (Kuczynski and Radler, 1982). In *S. cereviase* it has been speculated that malic enzyme could be involved in the anaplerotic supply of pyruvate during growth on ethanol and acetate (Boles *et al.*, 1998). These and other questions have arisen, particularly in the light of recent work that suggests that significant differences exist between the regulatory networks governing carbon metabolism in *S. cerevisiae* and *C. albicans* (Ramirez and Lorenz, 2007; 2009; Carman *et al.*, 2008). A complete understanding of the precise function and regulation of *C. albicans* carboxylate transporters will require quantitative knowledge of how responsive *C. albicans* is to changes in the activity of these transporters, how responsive these transporters are to changes in the environment, and how they interact with other proteins. These answers will be essential to have a detailed view of the metabolic flexibility of this important pathogen.

## Experimental procedures

### Yeast strains, plasmids and growth conditions

The yeast strains and the plasmids used in this work are listed in Tables 1 and 2 respectively. Cultures were maintained on YPD (Sherman, 1991). Yeast cells were grown at 30°C in minimal medium (yeast nitrogen base 0.67% w/v) supplemented with the appropriate requirements for prototrophic growth (Sherman, 1991) and containing carbon sources at the following concentrations: glucose (2% w/v: SD), lactic acid (0.5% v/v, pH 5.0), pyruvic acid (0.5% w/v, pH 5.0), succinic acid (1% v/v, pH 5.0) or malic acid (1% v/v, pH 5.0). Solid media was prepared adding agar (2% w/v) to the respective liquid media, and the pH for media containg carboxylic acids was always set either to 5.0 or 7.0. Cultures were harvested during the exponential phase (OD_640nm_ = 0.5). Yeast cells were grown under repression conditions in SD. For derepression conditions, glucose-grown cells were harvested, washed twice in ice-cold deionized water and inoculated into fresh minimal medium supplemented with the carbon source of choice.

### *C. albicans* mutant construction

The *C. albicans JEN2* gene was identified through homology to *ScJEN1* using the blast program. The two *JEN2* alleles in *C. albicans* strains RM1000 (*JEN1/JEN1*: Table 1) were inactivated sequentially with the *loxP-URA3-loxP* (LUL) and *loxP-HIS1-loxP* (LHL) markers (Dennison *et al.*, 2005). *jen2::LUL* and *jen2::LHL* disruption cassettes designed to delete the complete *JEN2* open reading frame were generated by PCR amplification with the primers CaJEN2-S1Fwd and CaJEN2-S2Rev (Table 3). The *jen2::LUL* cassette was transformed into *C. albicans* (Gietz and Woods, 1998), transformants selected on the basis of their uridine protrophy, and correct integration confirmed by diagnostic PCR with primers DCaURA3-1Rev and DCaJEN2lcFwd (Table 3) (Wilson *et al.*, 1999). The resultant heterozygote (*jen2::loxP-URA3-loxP/JEN2*) was then transformed with the *jen2::LHL* cassette, and accurate disruption of the remaining *JEN2* allele confirmed by PCR using primers DCaHIS1-1Rev and DCaJEN2lcFwd, and CaJEN2A1 and CaJEN2A2 (Table 3). This yielded the homozygous *null* mutant, CNV3 (*jen2::LUL/jen2::LHL*: Table 1). The genotypes of this mutant and all other mutants made in this study were confirmed by Southern blotting (not shown).

**Table 3:** Oligonucleotides used in this study.

Name	Sequence
CaJEN1Fwd	GCATGCTTAGCACTGGCACTGACGCGTATTAGTAAATAGACTTTAATTTAG CTTTTACCC
CaJEN1Rev	ATCGAATGCTTAACTGATCACGTCGACTTTTTAGTATTTGATTGAATT GAATTGGTTATAAGA
CaJEN2Fwd	GCATGCTTAGCACTGGCACTGACGCGTGAGCACTAACAATTAGTTGTACAGTTCAAAACTCCG
CaJEN2Rev	ATCGAATGCTTAACTGATCACGTCGACCCGTCTCATATTTCTAACCGATTGTGCCAGTGGCTC
DRPS10	GTGGTTGGAGCTTTGATG
DCaJEN2Rev	AGCCATGAGAGCCATCTC
CaJEN2A1	GGTGATACATATGGTAGA
CaJEN2A2	GTGATCCACATTGGATGG
CaJEN2-S1Fwd	GGATGAATTGAAACAATATTCCTGGCACGAAGTTCTTAATCCGTTTGAACCATTAGTGGA **CCTCTTCGCTATTACGCCAG**
CaJEN2-S2Rev	CGGCACCTCTGTTTTCAGGACCAATAAAAACAACAAACATCAAGTAAGCCAAAACAGCAC **GCAGATTACCCTGTTATCCCTA**
DCaURA3-1Rev	CTGCTCTCTCACTATAGGTC
DCaHIS1-1Rev	CGGTCTGGTAAATGATTGAC
DCaJEN2lcFwd	CCCAATACATCACATTAC
CaJEN2-DB1Fwd	CTAACCATAGAAATATTATGACTGCTGCTGATACTCATTCTATCACTAGTGCTGATG TTC **TTTTCCCAGTCACGACGTT**
CaJEN2-DB2Rev	CCACCAACACTTACTCTTTATGTTCAACTTCTGGTTTTTCCAAAGTCTTTTGAG TAATATTAC **TGTGGAATTGTGAGCGGATA**
CaJEN2-DB3Fwd	CATAATAGACACATTATTCGTCCACCAAAATTCACTTGGCCCGCTATTCGAAAATATGCC **TTTTCCCAGTCACGACGTT**
CaJEN2-DB4Rev	CTTCAAGATCAGAATCACCTCTGTCTTCCCTATCACTATCGTACGCACTGTATTCGTCATC **TGTGGAATTGTGAGCGGATA**
DminiURA3Rev	TAGAAGGACCACCTTTGATTGT
CaJEN2GFPFwd	GAAGAAGGTAATATTACTCAAAAGACTTTGGAAAAACCAGAA GTTGAACATAAAGAG **GGTGGTGGTTCTAAAGGTGAAGAATTATTC**
CaJEN2GFPRev	CACACACATACTATTTTAACAAATCATAAACCCATTTATTATCAAAATAAACTATACTTG **TCTAGAAGGACCACCTTTGATTG**
DJEN2GFP_Fwd	CTTGGTCAGTGGTGCCAA
GFP Rev	AACATCACCATCTAATTCAAC
JEN2_416For	GGGATCCAATATTATGACTGCTGCTGATACTCATTCTATC
JEN2_416Rev	GAAGCTTTTAGTGATGGTGATGGTGATGCTCTTTATGTTCAACTTCTGGTTTTTC
JEN2416ForREQ	AAAACACCAAGAACTTAGTTTCGACGGATTCTAGAACTAGTGGATCCAATATTATGACTGCTGCTGATACTCATTCTATC
JEN2416RevREQ	CATGACTCGAGGTCGACGGTATCGATAAGCTTTTAGTGATGGTGATGGTGATGCTCTTTATGTTCAACTTCTGGTTTTTC
URA3-dpl200fwd	TAAAACGACGGCCAGTGAAT
URA3-dpl200rev	ACCATGATTACGCCAAGCTC
RPS10	ACTAATTCTTCTCTTCAG
Ins CaJEN1	AAGTCTATTTACTAATACG
TDH promoter	ACAAGGCAATTGACCCACGCATGTATCTA
CYC Terminator	GAATGTAAGCGTGACATAACTAATTACATG

DNA sequences complementary to the sequences of pLUL and pLHL (S1/S2), pDDB57 (DB1/DB2/DB3/DB4) and pGFP-URA3 (F1/R1) are shown in bold.

The *JEN2 locus* was also disrupted in *C. albicans* strain CPK2 (*jen1/jen1*) to create the double *jen1jen2* mutant, CNV4 (Table 1). First, *ura3-* segregants of CPK2 were selected using 5-fluoroorotic acid (Boeke *et al.*, 1984). The first *JEN2* allele was disrupted using a cassette created by PCR amplification of the mini-*URA3* blaster (Wilson *et al.*, 2000) with primers CaJEN2-DB1 Fwd and CaJEN2-DB2 Rev (Table 3). *Ura3-* segregants were selected 5-fluoroorotic acid to create a *jen1/jen1, jen2/JEN2* heterozygote. The remaining *JEN2* allele in this strain was then disrupted using a second cassette created by PCR amplification of the mini-*URA3* blaster (Wilson *et al.*, 2000) with primers CaJEN2-DB3 Fwd and CaJEN2-DB4 (Table 3). Once again, *ura3-* segregants were selected 5-fluoroorotic acid to create a *jen1/jen1, jen2/jen2* double mutant, CNV4 (Table 1). Correct integration of the cassettes and loss of the wild-type *JEN2* allele were confirmed by diagnostic PCR with primers DCaJEN2lcFwd and DCaURA3-1Rev, DCaJEN2lcFwd and DminiURA3Rev, URA3-dpl200fwd and URA3-dpl200rev, and CaJEN2A1 and CaJEN2A2 (Table 3).

To reintroduce a functional *JEN1* gene into strains CPK2 and CNV4, the *JEN1* gene, plus approximately 2000 bp upstream and 600 bp downstream of its coding sequence were PCR amplified using primers CaJEN1Fwd and CaJEN1Rev (Table 3). The resulting PCR fragment was digested with SalI and MluI and ligated into CIp20 (Dennison *et al.*, 2005) to create CIp20-JEN1. This plasmid was then digested with StuI and integrated at the *RPS1* locus in *C. albicans* CPK2 and CNV4 (Murad *et al.*, 2000), thereby generating CNV2-2 and CNV4-2 (Table 1). Correct integration at the *RPS1* locus was confirmed by diagnostic PCR, using primers RPS10 and InsCaJEN1 (Table 3), and by Southern blot analysis. As controls the *C. albicans* strains CPK2, CNV3 and CNV4 were also transformed with the empty CIp20 plasmid. *JEN1* reintegration suppressed all *jen1/jen1* phenotypes, as expected (not shown). Multiple attempts were made to reintegrate *JEN2* into *jen2* mutant strains without success. It was not possible to clone the *JEN2* locus into several different types of *Escherichia coli* or *C. albicans* vectors using distinct PCR strategies or by using the Clonetech Cloning System (In-Fusion™ 2.0 Dry-Down PCR Cloning Kit; http://www.clontech.com). Additionally several attempts to clone PCR-amplified *JEN2* directly in *S. cerevisiae* and into *C. albicans* genomic locus proved unsuccessful. We conclude that some feature of the *JEN2* locus precludes its cloning using a range of standard procedures. This meant that it was not possible to restoring *JEN2* in the *jen2* mutant. In the absence of a *JEN2* reintegrant we compared the phenotypes of five independent *jen2* mutants. These *jen2* mutants displayed identical phenotypes under all the conditions tested.

### Construction of the *JEN2-GFP* fusion in *C. albicans*

To tag *C. albicans JEN2* at its 3-end, the GFP ORF was PCR amplified from pGFP-URA3 (Gerami-Nejad *et al.*, 2001) using primers CaJEN2GFPFwd and CaJEN2GFPRev (Table 3). The resultant PCR product was used to transform *C. albicans* RM1000, and transformants were screened for correct integration by diagnostic PCR using primers DJEN2GFPFwd and GFPRev (Table 3).

### Heterologous expression of *C. albicans JEN2* in *S. cerevisiae*

*Candida albicans JEN2* was cloned into the plasmid p416GPD (Mumberg *et al.*, 1995) by gap repair (Orr-Weaver and Szostak, 1983). To achieve this, the *JEN2* ORF was PCR amplified with primers JEN2416ForREQ and JEN2416RevREQ (Table 3). Both the plasmid and the PCR product were digested with BamHI and HindIII, purified from an agarose gel, and co-transformed into the *S. cerevisiae jen1* mutant (Table 1). Correct clones were identified by colony PCR using primers TDH promoter and CYC terminator (Table 3).

### DNA manipulations

Cloning, PCR amplification and Southern analysis were performed as described previously (Sambrook *et al.*, 1989; Dennison *et al.*, 2005).

### Transport assays

Cells incubated under derepressing conditions were harvested by centrifugation, washed twice in ice-cold deionized water and resuspended in ice-cold deionized water to a final concentration of about 25–40 mg dry wt. ml^−1^. 10 µl of yeast cell suspension were mixed in 10 ml of conical tubes with 30 µl of 0.1 M potassium phosphate, pH 5.0. After 2 min of incubation at 30°C in a water bath, the reaction was started by the addition of 10 µl of an aqueous solution of labelled carboxylic acid at the desired concentration and pH value, and stopped by dilution with 5 ml of ice-cold water. The reaction mixtures were filtered immediately through Whatman GF/C membranes, the filters washed with 10 ml of ice-cold water and transferred to scintillation fluid (Opti-phase HiSafe II; LKB FSA Laboratory Supplies, Loughborough, UK). Radioactivity was measured in a Packard Tri-Carb 2200 CA liquid scintillation counter. The following radiolabelled substrates were utilized: D,L-[U-^14^C] lactic acid, sodium salt (CFB97–Amersham Biosciences); [2,3-^14^C] succinic acid (NEN Life Science); and L-[1,4(2,3)-^14^C] malic acid (CFB42-Amersham). Nonspecific ^14^C adsorption to the filters and to the cells was determined by adding labelled acid after ice-cold water. Background values represented less than 5% of the total incorporated radioactivity. The transport kinetics best fitting the experimental initial uptake rates and the kinetic parameters were determined by a computer-assisted nonlinear regression analysis (GraphPAD Software, San Diego, CA, USA).

### Microscopy

*Candida albicans* and *S. cerevisiae* cells were examined with a Leica Microsystems DM-5000B epifluorescence microscope with appropriate filter settings. Images were acquired with a Leica DCF350FX digital camera and processed with LAS AF Leica Microsystems software.

### RNA isolation and qRT-PCR analysis

*Candida albicans* wild-type and mutant cells were grown in YNB media supplemented with 2% glucose, till an OD_640nm_ of approximately 0.5, and derepressed for 4 h in media containing different carbon sources, prepared as previously described. Total RNA was then isolated using the standard hot acidic phenol protocol. qRT-PCR was carried out to analyse the expression of *JEN1/JEN2* and *ACT1* in the conditions tested. qRT-PCR was performed in an LC480 equipment using the standard real-time PCR conditions. Ct values were transformed in expression values using standard curves made on a pool of samples. Both cDNA samples and RT-minus reactions (RNA samples treated in the same way but without the addition of the RT enzyme in the reverse transcription reaction) were analysed, and the level of cDNA was considered as the expression signal of each sample substracting the RT-minus signal. Finally, relative expression was calculated [(Gene of interest cDNA conc)/(calibrator cDNA conc)].

### Cell extracts and immunoblotting

Cells were grown in glucose 2% and derepressed for 4 h in media containing different carbon sources. Lactic acid derepressed cells were also subjected to pulses of 0.01, 0.05 and 0.1% glucose. Preparation of total protein extracts followed the NaOHTCA lysis technique (Volland *et al.*, 1994). Sample buffer was added to extracted proteins, heated at 37°C and resolved by SDS polyacrylamide gel electrophoresis in 10% acrylamide gels. The gels were run using a tricine buffer and transferred to PVDF membranes that were probed with monoclonal anti-GFP antiserum (Roche diagnostics) and anti-ACT1 (abcam, Cambrige, UK). Horseradish peroxidase-conjugated anti-mouse immunoglobulin G was used as the secondary antibody (Sigma, St Louis, MO USA) and was detected by enhanced chemiluminescence (ECL).

### *C. albicans* morphogenesis

Different environmental signals were used to induce hyphal development in *C. albicans.* To trigger hyphal development by serum yeast cells were grown in minimal medium containing 0.5% lactic acid, pH 5.0, and then they were incubated with 0, 10 or 20% FBS at 37°C, with agitation, for 3 h (Swoboda *et al.*, 1994). Cells were also platted in YPD agar supplemented with 10% FBS. To monitor the effect of pH, growth was also carried out in minimal medium, but at pH 6.5. The starter culture was divided into two flasks with lactic acid 0.5%, at pH 4.5 and pH 6.5. The culture at pH 6.5 was incubated at 37°C, whereas the one at pH 4.5 at 25°C, for 5 h. Finally, a nutrient limitation stress was imposed by incubating the cells with media containing N-acetylglucosamine (Mattia *et al.*, 1982). The cells were grown in Lee's medium at pH 4.5, 37°C, for several days, and then starved for 24 h, at 37°C. Afterwards, they were ressuspended in BSM medium with or without the addition of 4 mM GlcNAc. All flasks were then incubated for 5 h, at 37°C.

### *Ex vivo* models of *C. albicans* phagocytosis

Human Blood from several donors was collected by venepuncture using heparinized tubes. For blood killing assays, *C. albicans* cells were grown in the appropriate carbon source to an OD_640_ = 0.5, and then incubated with 100 µl of whole blood (100 cells: 100 µl of whole blood/neutrophils) at 37°C for 1 h. Cells were then plated on YPD and colony forming units (CFU) counted after 24 h, at 30°C. Neutrophils were isolated from human blood (Fradin *et al.*, 2005), *C. albicans* cells were washed with phosphate-buffered saline (PBS) and incubated with neutrophils in a 1:1 ratio. GFP fluorescence was measured after 2.5 h at 37°C (Barelle *et al.*, 2004). Control *C. albicans* cells were incubated with human plasma. Cultured murine RAW 264.7 macrophage (ECACC, Salisbury, UK), kindly provided by Leanne Clift (University of Aberdeen, UK), were diluted to 1 × 10^6^ cells ml^−1^ in supplemented DMEM media, plated in six well plates, and grown overnight at 37°C, under 5% CO_2_. *C. albicans* CPK20-5 and CNV30-5 (Table 1) were grown also overnight in minimal medium containing 0.5% lactic acid, pH 5.0 to an OD_640_ = 0.5, and then counted with a haemocytometer. Approximately 3 × 10^8^ cells were then added to the wells containing macrophages to give a *C. albicans* macrophage ratio of 3:1. Samples were then incubated for 3 h, at 37°C, under 5% CO_2_. Control cultures of *C. albicans* strains containing pICL1-GFP or the empty pGFP vector (Barelle *et al.*, 2004) were also incubated with macrophages (3:1). To provide a further control, *C. albicans* cells were incubated in macrophage growth medium (DMEM containing FBS and glutamine) with no macrophages. Cells were fixed, mounted and GFP quantified as described previously (Barelle *et al.*, 2004; 2006;).

### Murine model of systemic candidiasis

Female BALB/c mice (Harlan, UK) were handled and maintained according to the conditions specified by the Home Office (UK) regulations. As described previously (Barelle *et al.*, 2006), mice of approximately 6–8 weeks were infected with 2–6 × 10^4^*C. albicans* cells/g body weight by lateral tail vein injection. Actual levels of inoculation were assayed by viable plate counting. Fungal burdens and *in vivo* kidney sections were analysed after 3 or 4 days of infection (Fradin *et al.*, 2005). Kidneys were removed aseptically. Half of each kidney was used for determination of fungal burdens, and the other half was fixed in 4% paraformaldehyde. Fixed kidneys were embedded in Cryo-M-Bed (Bright, Huntingdon, UK) and flash-frozen. Sections (5 µm) were cut and stained with Calcofluor white to identify fungal cells (Barelle *et al.*, 2004). Images were generated at 461 nm (Calcofluor white staining), 516 nm (GFP) and 573 nm (Rhodamine as a control for GFP specificity). For the virulence assay strains were grown for 16 h in NGY medium (0.1% Neopeptone 0.4% glucose, 0.1% yeast extract), washed twice in sterile physiological saline and resuspended in saline to produce inocula of 5 × 10^4^ CFU g^−1^ bodyweight per mouse. Actual inocula were determined from viable plating of the inocula. Fungal burdens were determined for the kidneys and spleen of all mice. Organs were homogenized in 0.5 ml of saline and dilutions of the resulting homogenate plated onto Saboraud agar. Plates were incubated overnight at 35°C, and then colonies counted. Survival of mice was analysed by log rank/Kaplan–Meier statistics. Organ burdens were compared by the Mann–Whitney *U*-test.
